# Extubation and the Risks of Coughing and Laryngospasm in the Era of Coronavirus Disease-19 (COVID-19)

**DOI:** 10.7759/cureus.8196

**Published:** 2020-05-19

**Authors:** Karen S Sibert, Jennifer L Long, Steven M Haddy

**Affiliations:** 1 Anesthesiology and Perioperative Medicine, University of California, Los Angeles (UCLA) Health, Los Angeles, USA; 2 Head and Neck Surgery, University of California, Los Angeles (UCLA) Health, Los Angeles, USA; 3 Surgery and Perioperative Medicine, Veterans Administration Greater Los Angeles Healthcare System, Los Angeles, USA; 4 Anesthesiology, Keck School of Medicine of the University of Southern California, Los Angeles, USA

**Keywords:** laryngospasm, extubation, deep extubation, functional anatomy of the larynx, upper airway obstruction, partial airway obstruction, stridor, novel coronavirus, aerosol-generating procedures, covid-19

## Abstract

The coronavirus disease-19 (COVID-19) pandemic has prompted new interest among anesthesiologists and intensivists in controlling coughing and expectoration of potentially infectious aerosolized secretions during intubation and extubation. However, the fear of provoking laryngospasm may cause avoidance of deep or sedated extubation techniques which could reduce coughing and infection risk. This fear may be alleviated with clear understanding of the mechanisms and effective management of post-extubation airway obstruction including laryngospasm. We review the dynamic function of the larynx from the vantage point of head-and-neck surgery, highlighting two key concepts:

1. The larynx is a complex organ that may occlude reflexively at levels other than the true vocal folds;

2. The widely held belief that positive-pressure ventilation by mask can “break” laryngospasm is not supported by the otorhinolaryngology literature.

We review the differential diagnosis of acute airway obstruction after extubation, discuss techniques for achieving smooth extubation with avoidance of coughing and expectoration of secretions, and recommend, on the basis of this review, a clinical pathway for optimal management of upper airway obstruction including laryngospasm to avoid adverse outcomes.

## Introduction and background

The advent of the novel severe acute respiratory coronavirus 2 (SARS-CoV-2) and the ensuing coronavirus disease-19 (COVID-19) pandemic have transformed routine intubation and extubation of the trachea in affected patients into hazardous procedures during which the highly contagious virus has the potential to become aerosolized. Current recommendations for extubation of COVID-19 patients include performance in an airborne isolation room and the advice that “the endotracheal tube be removed as smoothly as is feasible” to avoid coughing and expectoration of virus-laden secretions [1]. For non-emergent operating room cases, extra precautions to minimize aerosolization of virus-containing droplets are warranted in case patients might be in an early, asymptomatic stage of infection. This advice has prompted renewed interest in the technique of deep extubation among American anesthesiologists and other physicians, many of whom (as opposed to colleagues in the United Kingdom) are accustomed to performing extubation only when a patient is fully awake and able to follow commands.

Reluctance to perform extubation in a patient who demonstrates adequate spontaneous breathing but is not fully awake may be attributed to concern that the patient could develop laryngospasm during emergence from sedation or general anesthesia. When trainees in anesthesiology are queried about their level of knowledge concerning the diagnosis and treatment of laryngospasm, the authors frequently have encountered the following responses:

1. Laryngospasm is defined as sudden closure of the vocal cords;

2. Laryngospasm may be “broken” with positive-pressure ventilation by mask;

3. Laryngospasm results from “Stage 2” anesthesia during induction or emergence.

Unfortunately, none of these statements is fully accurate, and the consequences of misunderstanding may be dire. Failure to diagnose and successfully treat laryngospasm or any other form of airway obstruction may result in hypoxemia, bradycardia, negative-pressure pulmonary edema, and cardiac arrest. Particularly in pediatric patients, laryngospasm is considered to be a significant factor in perioperative morbidity and mortality, cited as the proximate cause in 8% of pediatric cardiac arrests related to anesthesia in a recent major review [2]. 

The lack of clear understanding of the mechanics of laryngospasm is at the heart of the problem. While the technical definition of laryngospasm is generally understood as prolonged involuntary occlusion of the glottis, this narrow definition neglects the complex functional anatomy of the upper airways. Airflow may be blocked at levels other than the vocal cords, and positive-pressure ventilation paradoxically may worsen rather than alleviate laryngeal occlusion. Understanding the functional anatomy of the larynx is critical to the management of dynamic airway occlusion including laryngospasm. This paper reviews laryngeal function and outlines differential diagnostic considerations for acute airway obstruction following extubation. We review methods to achieve smooth extubation without coughing or agitation, and offer a clinical pathway to diagnose and terminate upper airway obstruction including laryngospasm promptly should it occur. The mastery of technique for successful and smooth extubation has the potential to reduce the risk of contracting COVID-19 to the benefit of operating room and intensive care unit personnel.

## Review

Dynamic function of the larynx

**Figure 1:**
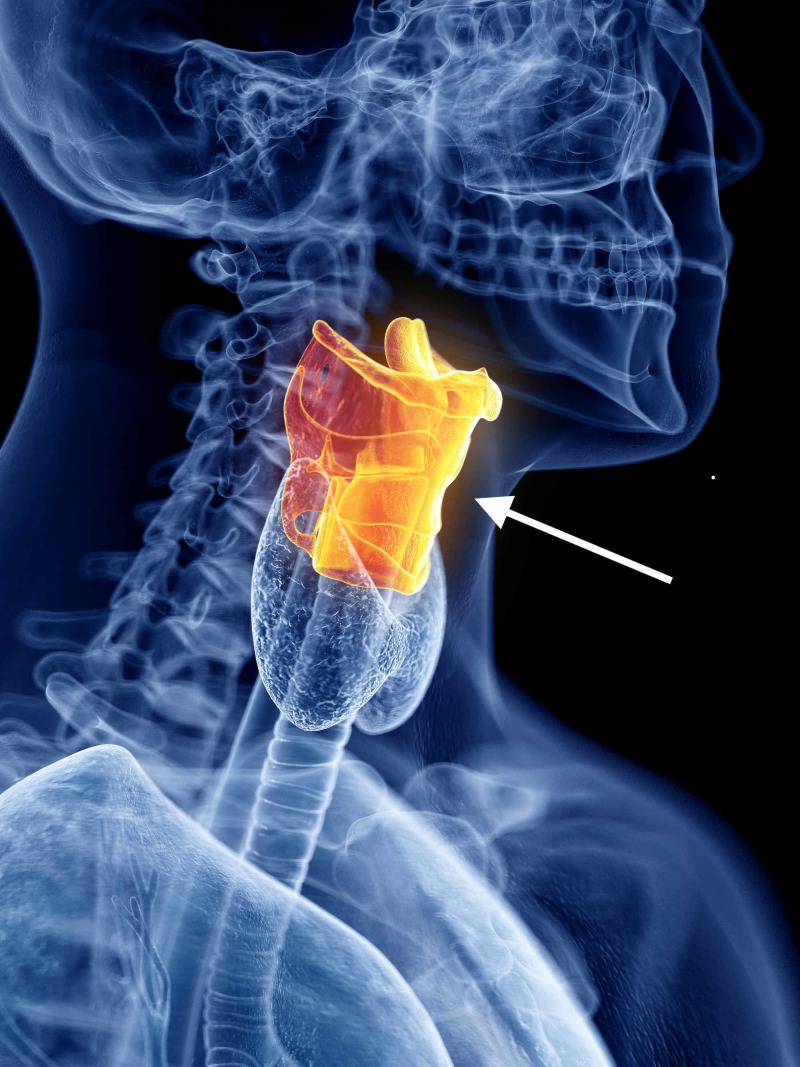
Digital rendering of the larynx The larynx is a complex functional organ that serves as the guardian of the airway, and is located between the third and sixth cervical vertebrae. (Artist: Sebastian Kaulitzki, via Shutterstock, Inc., with permission.)

The larynx is a complex functional organ that serves as the guardian of the airway, allowing air to pass and protecting against the incursion of secretions, food, and airway irritants. In adults, it measures approximately 4 cms in craniocaudad dimension and 3 cms in transverse diameter, and is located at the level of the third through sixth cervical vertebrae, at the entry to both the trachea and the esophagus, as illustrated in Figure 1.

**Figure 2:**
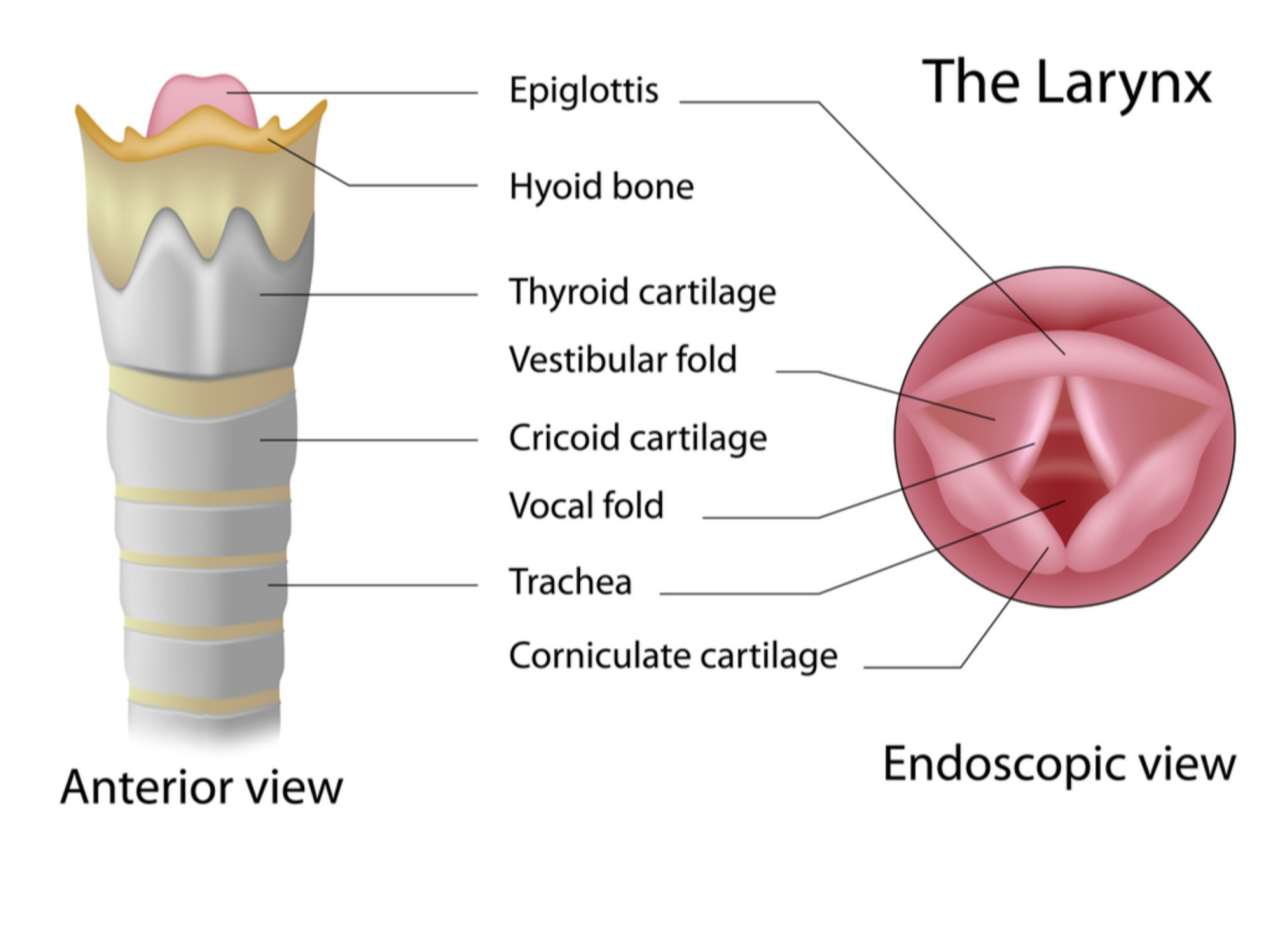
Anatomy of the larynx Laryngeal closure may occur at three levels from superior to inferior: the laryngeal inlet between the hyoid bone and the thyroid cartilage, the vestibular folds or false cords, and at the glottis between the vocal folds. (Image by Alila Medical Media, via Shutterstock, Inc., with permission.)

The larynx evolved in mammals to separate the respiratory tract from the alimentary tract, when both share a common upper aerodigestive pathway [3]. Its primary purpose is to prevent aspiration of food into the trachea during swallowing; phonation evolved much later as a secondary benefit. The thyroid, cricoid, and paired arytenoid cartilages provide the skeletal framework, connected and supported by intrinsic laryngeal muscles, ligaments, and synovial joints. The paired vocal folds span the distance between the arytenoid cartilages posteriorly and the anterior angle of the thyroid cartilage. The term vocal “fold” is more precise than vocal “cord” because the lateral edge of the tissue is not free. Above these true vocal folds are the structures colloquially known as the false cords, also called the vestibular or ventricular folds. The medial space between the true vocal folds is the glottis, shown in Figure 2.

The larynx has a complex and variable system of motor and sensory nerve fibers derived from the superior and recurrent laryngeal nerves, communicating via brainstem-mediated reflex arcs. Significant variation in nerve anastomosis patterns has been observed in human cadaver studies, which may account for some of the biological differences underlying susceptibility to laryngospasm [4]. The sensory fibers are most heavily distributed in the laryngeal inlet and epiglottis, but pharyngeal plexus sensory fibers also anastomose with laryngeal motor nerves in a reflex arc [5,6]. Closure of the larynx can occur at three different anatomic levels, at the vocal folds and at two additional levels superior to the vocal folds:

1. The aryepiglottic folds, located at the entrance of the larynx and extending from the lateral borders of the epiglottis to the arytenoid cartilages, exhibit sphincteric closure to appose the epiglottis;

2. The vestibular folds or false cords compress and may completely block the supraglottic airway;

3. The vocal folds close when the lateral cricoarytenoid, thyroarytenoid, and interarytenoid muscles contract.

To protect the airway during swallowing, the intrinsic muscles close the larynx at all three levels, superior to inferior - the laryngeal inlet including the epiglottis, the false cords, and the true vocal cords. A similar but stronger laryngeal adductor reflex acts to protect the lower airways from noxious environmental stimuli such as inhaled smoke or chemicals [7]. In the setting of intensive care and/or anesthesia, this reflex glottic closure may occur following innocuous stimuli: secretions irritating the larynx, a rapid inflow of cold dry air as with an “oxygen flush” from the anesthesia circuit, or a foreign body such as an endotracheal tube.

The reflex closure of the larynx is a primitive, hard-wired survival mechanism, and the vocal folds function as the last line of defense with a valvular effect to prevent entry of foreign matter. While the true vocal folds are passively relaxed and open during quiet breathing, it has been known since the 19th century that their tight adduction prevents the entrance of air or any other substance [8]. The true vocal cords abduct in response to pressure from below, as in coughing or laughing, not to pressure from above. The external pressure required to overcome the valvular closure of the true vocal cords approaches 140 mmHg or 190 cmH_2_O in adults and up to 50 cmH_2_O in infants and children [9,10]. To put these numbers in perspective, the default high-pressure setting on many anesthesia machines is 40 cmH_2_O and the high-pressure safety valve on the ventilator is typically set to release at 75 cmH_2_O. It would be difficult to obtain a seal with a conventional facemask sufficient to generate such peak pressures with manual ventilation. As Petcu and Sasaki have observed, “This protective arrangement can be especially appreciated by the difficulty experienced with overcoming laryngospasm by positive-pressure ventilation” [9]. The powerful closure of the vocal folds in laryngospasm may continue well beyond the cessation of the stimulus that provoked it [9,11].

In the classic 1956 article, “The Etiology and Treatment of Laryngeal Spasm”, Fink described reflex laryngeal closure in terms of the larynx functioning as “a shutter and a ball valve” [12]. The “shutter” closes by adduction of the true vocal folds, while the “ball valve” is created by closure of the false cords and by the approximation of the soft tissues of the laryngeal inlet above, extending from the hyoid bone to the notch of the thyroid cartilage. As a result, “the entire larynx is firmly closed,” Fink concluded. “Ball-valve closure of the larynx may constitute a serious emergency. Such a spasm cannot be broken by bag pressure through a mask. Forced inflation of the pharynx merely distends the piriform fossas on either side of the larynx and presses the aryepiglottic folds more firmly against each other.”

The role of positive-pressure ventilation by mask

If closure of the larynx resistant to positive-pressure ventilation has been eloquently described in the anesthesiology and otorhinolaryngology literature for many years, why have generations of anesthesiologists persisted in the belief that positive-pressure ventilation will “break” laryngospasm? Part of the answer is that standard anesthesiology references continue to advocate positive-pressure ventilation by mask as the first step in the management of laryngospasm even while acknowledging that often it fails [10,13]. Another part of the answer may be that eventually the patient in laryngospasm - pediatric or adult - will develop hypercarbia which depresses adductor activity, as will hypoxemia if the partial pressure of oxygen in arterial blood (PaO_2_) declines to less than 50 mmHg [11,14]. If hypoxemia and hypercarbia are the result of futile attempts at mask ventilation, these attempts may be falsely credited with success in “breaking” laryngospasm. However, severe hypoxemia and hypercarbia cannot be viewed as desirable routes toward rescue, and may lead rapidly to bradycardia and cardiac arrest in infants and young children. In adults, negative-pressure pulmonary edema may result from a patient’s strenuous efforts to breathe during prolonged airway obstruction [15-18].

Confusion also results from a tendency to conflate stridor and laryngospasm. Some writers refer to “partial laryngospasm”, which may be more accurately described as inspiratory stridor [19]. This phenomenon may be seen with vocal cord dysfunction or damage. In anesthesiology practice, stridor may be encountered immediately after extubation while the laryngeal muscles are still in a state of relaxation from the residual effects of inhalation anesthesia or sedatives, and the opening of the airway is incomplete. The Bernoulli effect causes increased velocity of airflow through the narrowed laryngeal inlet and glottis during spontaneous inspiration, resulting in a musical or whistling inspiratory stridor or “crowing”. This effect typically will dissipate on its own as recovery from anesthesia continues. However, it may be improved with gentle positive-pressure assistance to increase airway diameter and neutralize the Bernoulli effect [12]. In contrast to stridor, complete laryngospasm is silent with no movement of air between the closed larynx and the lungs, and attempts at positive-pressure ventilation may serve only to aggravate the problem [9,12].

Risk factors for laryngospasm

Anesthesiologists rarely encounter laryngospasm outside the perioperative setting, which explains the prevalent misconception that it occurs only upon induction or extubation. However, laryngospasm may occur as a result of gastroesophageal reflux, severe hypocalcemia, Vitamin D deficiency, Parkinson’s or other neurodegenerative diseases, and inhalation of noxious fumes. Laryngospasm may also occur as a manifestation of reactive airways disease [20,21]. The risk factors for laryngospasm in pediatric anesthesiology have been comprehensively reviewed, with the list including younger age, recent upper respiratory illness, asthma, and procedures involving the airway or pharynx [14,19].

Aspiration of gastric contents or oropharyngeal secretions may provoke laryngospasm, and may cause respiratory complications including pneumonitis and pneumonia. The twin fears of aspiration and laryngospasm are the two reasons most often cited by anesthesiologists as reasons to extubate patients only when they are fully awake [22]. Certainly, appropriate patient selection is critical to prevent aspiration whenever the airway is unprotected, whether the setting is induction of general anesthesia or deep extubation at procedure end (appropriate patient selection is equally important in the decision to employ a laryngeal mask airway, or to provide deep sedation with an unprotected airway for endoscopy, angiography, or surgery under monitored anesthesia care). Contraindications include but are not limited to a non-fasted state, achalasia, difficult airway, gastric outlet or bowel obstruction, gastroparesis, ileus, impaired swallowing, morbid obesity, and pregnancy. Of note, the use of sugammadex instead of neostigmine to reverse muscle relaxation was associated with a 30%-50% lower risk of postoperative pneumonia and respiratory failure in a recent large multi-center study, suggesting that incomplete reversal of muscle relaxant may play a larger role than previously recognized in pulmonary complications including aspiration [23].

The general consensus among anesthesiologists is that poorly managed extubation may provoke laryngospasm as the removal of the endotracheal tube is a deeply noxious stimulus [11,24]. It is an article of faith in anesthesiology education that extubation should be performed with the patient either deeply anesthetized or fully awake [10]. Yet in practice, many experienced physicians will extubate before patients have fully emerged from sedation or anesthesia [22]. Frost and colleagues have described success with the technique of “sedated extubation,” in which the patient demonstrates return of protective airway reflexes prior to extubation but is not reacting to the endotracheal tube to the point of gagging or coughing [25]. There are no data to support the hypothesis that one method of extubation is conclusively better than another in preventing laryngospasm. In pediatric patients, a recent metanalysis concluded that deep extubation reduces the overall risk of airway complications, and that no difference was observed in the risk of laryngospasm or breath-holding regardless of extubation technique or airway device [26]. The major differentiating factor may be operator expertise in managing the airway.

How important is “Stage 2” anesthesia in the development of laryngospasm, either during induction or during extubation and emergence from anesthesia? First, it must be noted that “Stage 2” is defined as a “delirium” or “excitement” stage of light anesthesia, first described by Guedel in 1927 while using diethyl ether, and characterized by rapid eyeball activity, swallowing, variable respiration, and potential vomiting [27]. It is rare to see an “excitement” phase with the balanced techniques of anesthesia in common use today. During inhalation induction, it is unwise to attempt to instrument the airway too early as the patient will react by coughing or moving, but overpressure with a high inspired concentration of sevoflurane quickly bypasses any excitement stage on the way to achieving the deeper stage of surgical anesthesia [10]. During emergence, today’s anesthetics balance the use of intravenous sedatives and analgesics, less-soluble inhalation agents, antiemetic medications, and multimodal analgesia. The development of new anesthetic agents in the second half of the 20th century effectively eliminated diethyl ether - and with it, a prolonged excitement phase during induction or emergence - from modern practice. Extubation of a deeply anesthetized or sedated patient today routinely produces a smooth emergence without laryngospasm or an excitement phase that would be consistent with the traditional description of “Stage 2” anesthesia. Excitement and uncontrolled behavior are more likely to ensue from awake extubation if vigorous suctioning, physical stimulation, and loud verbal commands precede removal of the endotracheal tube [25].

There are many situations where coughing or “bucking” is undesirable, either during emergence from anesthesia or weaning from mechanical ventilation in the intensive care unit. Poorly controlled waking and emergence could jeopardize the integrity of abdominal wound closure, or increase the risk of hematoma and airway compromise after thyroidectomy, carotid endarterectomy, or head-and-neck surgery. Coughing and expectoration of secretions that become aerosolized during extubation could increase the risk to all nearby personnel of contracting COVID-19 or other respiratory infections [1]. Control of blood pressure and heart rate is demonstrably easier to achieve with a well-controlled sedated or deep extubation [28,29].

The moment of greatest risk of upper airway obstruction, whether from laryngospasm or other causes, is the same for deep or awake extubation: it occurs when the endotracheal tube is removed [13,24]. Even when a patient does not react with a significant heart rate increase or cough during extubation, a moment of breath-holding or transient upper airway obstruction may follow, which must be managed with close observation and appropriate upper airway support until quiet, regular breathing resumes. The administration of intravenous lidocaine prior to extubation has been demonstrated to decrease airway irritability without depressing respiration or delaying emergence [30-32]. The selective use of propofol, fentanyl, remifentanil, hydromorphone, tramadol, ketamine, dexmedetomidine, or magnesium may smooth the course of extubation, whether the intent is to extubate the trachea with the patient awake, sedated, or deeply anesthetized [13,28,29,33,34]. Regardless of specialty, the physician's experience and finesse using any of these medications as adjuncts to event-free emergence and extubation may be the best predictors of success.

Post-extubation respiratory distress

Laryngospasm in clinical practice is a diagnosis of exclusion made when airway obstruction is difficult to alleviate. It is not possible to diagnose laryngospasm with certainty based on external observation alone. Many physicians have been called to an operating room, post-anesthesia care unit, or intensive care unit to help manage respiratory distress in a struggling patient. Rapid appreciation of the clinical presentation and a clear history of the immediately preceding events are critical for accurate diagnosis and timely rescue. Such cases may represent laryngospasm, or they may reflect supraglottic airway obstruction at the level of the tongue or the soft palate, as in obesity or obstructive sleep apnea. Other possible clinical diagnoses, summarized in Table 1, include airway edema, tracheomalacia, vocal cord paralysis, tetany due to hypocalcemia after parathyroidectomy, bronchospasm, pneumothorax, or inadequate reversal of muscle relaxant.

**Table 1:** Differential diagnosis of post-extubation respiratory distress *Causes of muscle weakness include aminoglycoside antibiotics, hypermagnesemia, inadequate reversal of muscle relaxant, and preexisting diseases including myopathies, prior stroke, myasthenia gravis, or Eaton-Lambert syndrome

Post-surgery	Cardiopulmonary	Upper Airway Obstruction
Cervical hematoma, swelling	Aspiration	Airway or pharyngeal swelling, including tongue, uvula
Hypocalcemia, tetany after parathyroidectomy	Bronchospasm	Laryngospasm
Paralyzed vocal cord	Congestive heart failure	Muscle weakness*
Pneumothorax	Foreign body in airway	Obesity
Tracheomalacia after thyroidectomy	Negative-pressure pulmonary edema	Obstructive sleep apnea

The successful management of upper airway obstruction hinges on prompt recognition and action. In a patient of any age who is making vigorous effort to breathe spontaneously but is not achieving effective air exchange, immediate attempts to ventilate with positive pressure by mask may only make airway closure worse and risk inflating the stomach [9,12]. If laryngospasm is suspected, the better initial step is to proceed with maneuvers to open the airway, as Fink recommends, beginning with “strong pressure behind the angles of the jaw” and optional insertion of a supraglottic airway [12,35]. An additional option is to apply firm upward pressure as described by Larson at the “laryngospasm notch” just below the earlobe - bounded anteriorly by the condyle of the mandible, posteriorly by the mastoid process, and superiorly by the base of the skull - while lifting the mandible and delivering oxygen by mask without positive pressure, as demonstrated in Video 1 [36,37,38].

**Video 1:** The Larson maneuver to alleviate laryngospasm, New England Journal of Medicine video [38]

**Figure 3:**
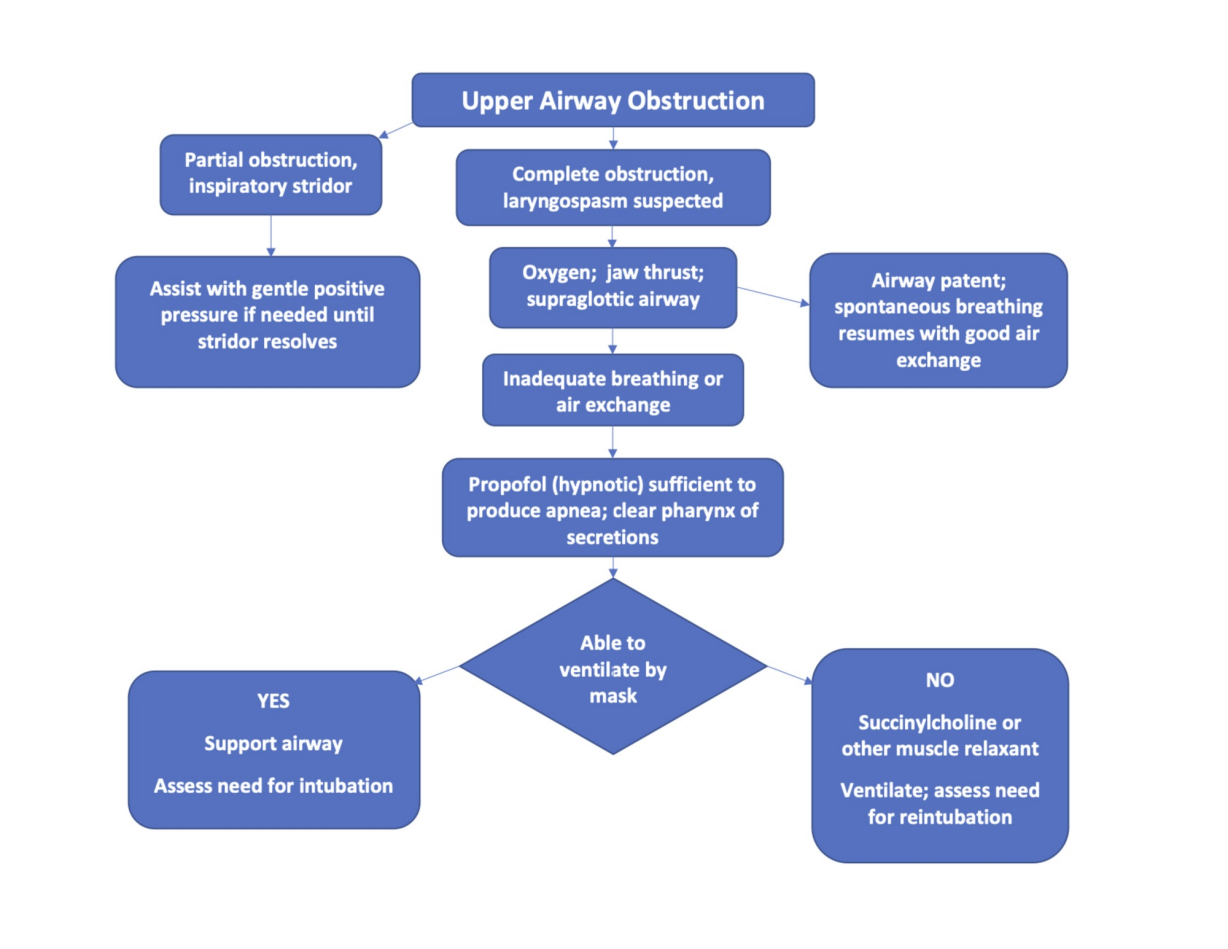
Clinical management of upper airway obstruction The successful diagnosis and management of upper airway obstruction depend on rapid analysis of whether the obstruction is complete or partial, and application of appropriate stepwise actions to open the airway and ensure ventilation.

Should adequate spontaneous air exchange not result within a few breaths, it is crucial to administer adequate sedation without delay in order to take control of the airway and lower the risk of hypoxemia and/or negative-pressure pulmonary edema. In a vigorous adult, powerful negative inspiratory force against an obstructed airway can generate pulmonary edema in only two or three breaths [15-18]. Delaying the decision to take definitive action increases the risk of serious complications with each passing moment. The key is to induce sleep and relaxation, even if accompanied by brief apnea. Propofol alone may suffice if the patient has intravenous access, though a muscle relaxant such as succinylcholine may be required for the treatment of refractory cases. For infants and young children without intravenous access, intramuscular succinylcholine may be necessary to permit successful mask ventilation [19]. As the larynx relaxes and the airway opens, any secretions can be cleared, mask ventilation becomes possible, and the physician can decide calmly whether or not to intubate or reintubate, as outlined in the algorithm, Figure 3. Often, if recognition of laryngeal occlusion is prompt and successful treatment is employed, hypoxia, hypercarbia, and intubation may be avoided.

## Conclusions

Review of the literature does not support the pervasive belief that positive-pressure ventilation can "break" laryngospasm, or that laryngospasm is defined exclusively as reflexive closure of the true vocal folds. It supports the conclusion that smooth extubation without active coughing - whether awake, sedated, or deep - may be accomplished with care but without fear once there is clear understanding of the functional anatomy of the larynx and of dynamic airway obstruction, which can occur at levels of the pharynx and larynx cephalad to the true vocal folds. There are many approaches to achieving smooth extubation with a variety of adjunct medications. Should upper airway obstruction occur after extubation, the steps toward successful management are straightforward. Even true laryngospasm should never be more than a nuisance, should rarely require positive-pressure ventilation or reintubation, and with vigilance should never result in tragedy. Smooth, well-controlled extubation reduces coughing, expectoration, and the unnecessary exposure of operating room and intensive care personnel to aerosolized, potentially infectious, airway secretions.
